# DNA polymerase β suppresses somatic indels at CpG dinucleotides in developing cortical neurons

**DOI:** 10.1073/pnas.2506846122

**Published:** 2025-08-13

**Authors:** Noriyuki Sugo, Arikuni Uchimura, Risa Matsumoto, Hiro Nakayama, Shota Fujimoto, Saya Mizuno, Mayumi Higuchi, Masaaki Toshishige, Yasunari Satoh, Sayaka Wakayama, Teruhiko Wakayama, Takeshi Yagi

**Affiliations:** ^a^Graduate School of Frontier Biosciences, Osaka University, Suita, Osaka 565-0871, Japan; ^b^Department of Molecular Biosciences, Radiation Effects Research Foundation, Hiroshima 732-0815, Japan; ^c^Advanced Biotechnology Center, University of Yamanashi, Kofu, Yamanashi 400-8510, Japan

**Keywords:** neurogenesis, mutagenesis, DNA repair, CpG demethylation

## Abstract

Somatic mutations in cortical neurons have been implicated in psychiatric disorders. While endogenous DNA damage and repair errors are potential contributors to these mutations during development, the underlying mutagenic mechanism remains unclear. Here, we investigated somatic mutations in immature cortical neurons using mouse somatic cell nuclear transfer-derived embryonic stem cells and whole-genome sequencing. Insertions and deletions (indels) were commonly observed in both repeat and nonrepeat sequences in wild-type cells. The loss of DNA polymerase β (Polβ), an enzyme involved in gap-filling during base excision repair and Ten-Eleven Translocation (TET)-mediated active DNA demethylation, in neural progenitor cells increased indel frequency by ~ninefold at cytosine-phosphate-guanine (CpG) dinucleotides and raised the frequency of structural variants by ~fivefold. These mutations were enriched in neuronal genes, leading to frameshift mutations, amino acid insertions/deletions, and the gain and loss of CpG sites in regulatory regions. Our findings suggest that Polβ preferentially repairs DNA lesions generated at CpG sites by TET-mediated active demethylation, thereby suppressing the mutagenesis that accompanies neuronal gene activation during cortical development.

## Significance

Proper brain development relies on the precise activation of neuronal genes. When this process fails, harmful changes can lead to mental illness. To activate key genes, developing brain cells must remove chemical tags called DNA methylation marks. However, this erasure can accidentally damage the DNA itself unless the DNA repair enzyme DNA polymerase β (Polβ) quickly fixes it. We found that such damage occurs most often at CpG sites—short DNA motifs that regulate many neuronal genes—and increases mutation frequency by ~ninefold, potentially disrupting neuronal function. These findings reveal a hidden cost of gene activation in the brain and help explain how DNA damage during development may contribute to the origins of psychiatric conditions.

Single-cell whole-genome sequencing (WGS) of postmortem human brain samples has revealed somatic mosaicism among cortical neurons (1–4). Somatic mutations, including single-nucleotide variants (SNVs), small insertions and deletions (indels), structural variations (SVs) such as copy number variations, and mobile element transpositions, have been observed in cortical neurons from developmental stages and have been implicated in neurodevelopmental and psychiatric disorders (5–8). Understanding the mechanisms behind these mutations is crucial for elucidating their role in disease pathogenesis. Previous studies suggest that replication stress-induced DNA double-strand breaks (DSBs) and error-prone DNA repair via nonhomologous end-joining (NHEJ) contribute to SVs in both mouse and human neural progenitors (9, 10). However, the mechanisms of endogenous DNA damage and mutagenesis in the developing cortex remain poorly understood (11, 12).

X-family DNA polymerase β (Polβ) plays a pivotal role in single nucleotide gap-filling during short-patch base excision repair (BER) (13). In addition, thymine DNA glycosylase (TDG)–dependent BER is also involved in TET-mediated active DNA demethylation process that replaces 5-methyl-cytosine (5mC) with cytosine at CpG dinucleotides (14). Polβ-deficient mice exhibit increased apoptosis in immature neurons due to PARP1 and p53 activation in the developing nervous system, leading to lethality immediately after birth (15–17). Our previous research demonstrated that Polβ loss increases single-strand breaks (SSBs) and DSBs in cortical neural progenitors and developing neurons during the period of neurogenesis and postnatal development. Furthermore, TET activity influences the extent of DSB formation (18, 19), raising the possibility that active DNA demethylation contributes to mutagenesis in neurons.

Although mutations in the Polβ gene have been reported in various cancers (20–22), how the loss of Polβ affects mutagenesis remains controversial. Both loss-of-function and gain-of-function studies demonstrate that Polβ exhibits a mutator phenotype in response to monofunctional alkylating agents (23, 24). Under physiological conditions in vivo, the effects of Polβ appear to be cell type- and environment-dependent (25–28). Despite extensive research on Polβ in cancer and DNA repair, its role in neuronal mutagenesis during cortical development remains unclear. Therefore, understanding the relationship between the Polβ-mediated process and neuronal mutagenesis in the developing cortex is critical.

This study aimed to elucidate the mutagenic mechanism following cortical neurogenesis. We investigated somatic mutations in wild-type and Emx1-Cre-driven Polβ-deficient immature excitatory cortical neurons during late embryonic stages. To detect rare somatic mutations in neurons, we used somatic cell nuclear transfer (SCNT) technique. Embryonic stem (ES) cell lines, specifically nuclear-transferred ES (ntES) cells, were established from neuronal nuclear-transferred embryos (29, 30). This approach allows precise replication of the original neuronal genome in proliferating ntES cells instead of in vitro single-cell whole-genome amplification. Their WGS and variant analysis enable the robust detection of de novo variations within individual neurons (31, 32). We found that indels at CpG dinucleotides are particularly prominent in Polβ-deficient clones, resulting in the gain and loss of CpG sites. The indel sites were enriched in regulatory and coding regions of neuronal genes. These results suggest that Polβ repairs DNA lesions generated at CpG sites by TET-mediated active demethylation and thereby suppresses somatic mutagenesis during neuronal gene activation in cortical development.

## Results

### WGS of Cortical Neuron Nuclei-Derived ntES Cells.

**Fig. 1.**
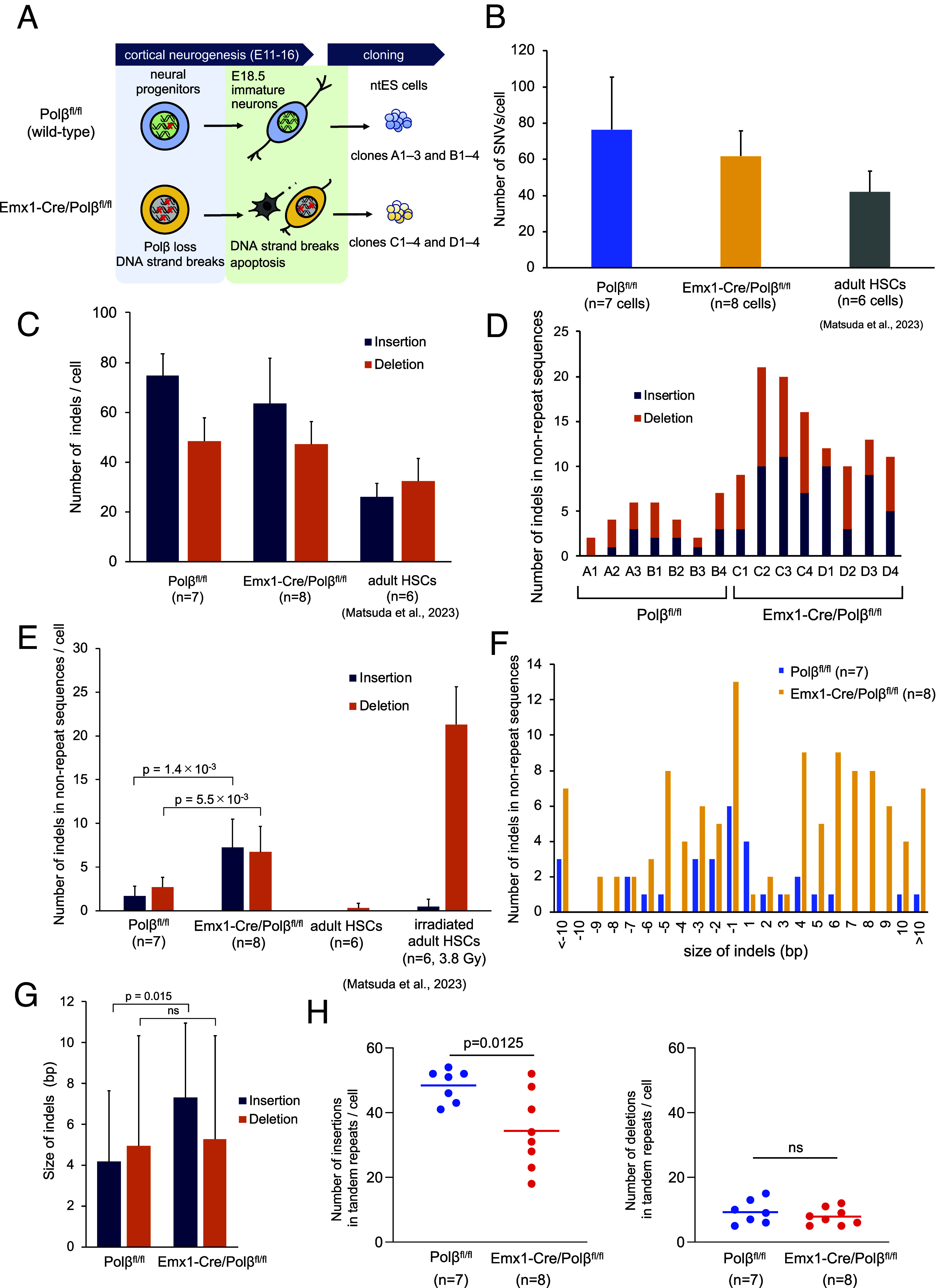
Somatic SNV and indel analyses in cortical neurons reprogrammed via somatic cell nuclear transfer. (*A*) Experimental design to investigate mechanisms of somatic mutations during neurogenesis and neuronal differentiation in the cerebral cortex using Emx1-Cre/Polβ^fl/fl^ and Polβ^fl/fl^ mice. (*B*) Mean number of somatic SNVs in Emx1-Cre/Polβ^fl/fl^, Polβ^fl/fl^ ntES cell lines, and adult HSCs (33). (*C*) Mean number of somatic insertions and deletions in Emx1-Cre/Polβ^fl/fl^, Polβ^fl/fl^ ntES cells, and adult HSCs (33). (*D*) Number of indels in nonrepeat sequences in each Emx1-Cre/Polβ^fl/fl^ and Polβ^fl/fl^ ntES cell. (*E*) Mean number of insertions and deletions in nonrepeat sequences of Emx1-Cre/Polβ^fl/fl^, Polβ^fl/fl^ ntES cells, and adult HSCs (33). (*F*) Distribution of indel lengths in nonrepeat sequences of Emx1-Cre/Polβ^fl/fl^ and Polβ^fl/fl^ ntES cells. (*G*) Mean indel size in nonrepeat sequences of Emx1-Cre/Polβ^fl/fl^ and Polβ^fl/fl^ ntES cells. (*H*) Number of indels in tandem repeats in each Emx1-Cre/Polβ^fl/fl^ and Polβ^fl/fl^ ntES cells.

To investigate low-frequency mutagenesis occurring during neurogenesis and neuronal differentiation, we performed SCNT by transferring donor nuclei of cortical neurons, dissociated from E18.5 Emx1^Cre/+^Polβ^fl/fl^ (Emx1-Cre/Polβ^fl/fl^) and littermate embryos, into wild-type enucleated oocytes. We then established seven ntES cell lines (clones A1-3 and B1-4) from two wild-type embryos and eight ntES clones (clones C1-4 and D1-4) from two Emx1-Cre/Polβ^fl/fl^ embryos (Fig. 1*A* and *SI Appendix*, Fig. S1*A*). In Emx1-Cre/Polβ^fl/fl^ embryos, Polβ is specifically disrupted in neural progenitors of the dorsal telencephalon before the onset of neurogenesis (19). We conducted whole genome sequencing for each clone (coverage range: 34× to 59×). The sequencing depth analysis revealed an aneuploidy event in clone C3, specifically the loss of the Y chromosome. In clone B3, a segment of chromosome 10 (from position 6,148,756 to the telomere) was deleted and subsequently duplicated.

De novo SNV and indel mutations were called using the Genome Analysis Toolkit (GATK) HaplotypeCaller (34). De novo mutation candidates were identified based on variant allele frequency (VAF) (31, 32). If a heterozygous mutation occurs in a neuron, the expected value of VAF in ntES cells is 50%. To exclude mutations arising during ntES cell establishment and culturing, we applied a threshold. Unique mutations were defined as those with a VAF >30% in a clone and <1% in sister clones, taking sequence coverage heterogeneity into account (*SI Appendix*, Fig. S1*B*). In addition to unique mutations in each clone, multiple shared mutations were also observed among some sister clones (*SI Appendix*, Fig. S1 *C* and *D*). The observed SNV counts in the seven wild-type cells (E18.5: 57 to 146, 82 ± 31.2/cell) are largely consistent with those reported in seven ntES cells (3 to 18 wk old: 50 to 112, 68.5 ± 22.9/cell) derived from mouse juvenile and adult olfactory neurons (32). In contrast, the SNV count in mouse adult clonal hematopoietic stem cells (HSCs; 16 wk old: 55 ± 16.4/cell), analyzed in the present study, was slightly lower (33). Moreover, compared to mouse postzygotic cells during early embryogenesis (1.0 SNVs per cell division) (31), cortical neurons—which undergo approximately 11 cell divisions during neurogenesis—exhibited a higher mutation rate (5 to 13 SNVs per cell division) (35). This higher rate is consistent with that observed in olfactory neurons (4 to 10 SNVs per cell division) (32) and is similar to that reported for human clonal neural progenitor cells (8.6 SNVs per cell division) (36).

In the following comparative analysis of Polβ-deficient and wild-type cells, shared mutations were excluded since their origin could not be definitively attributed to neurogenesis rather than early embryogenesis (31). The number of unique SNVs ranged from 46 to 140 in the seven wild-type clones and from 34 to 85 in the eight Polβ-deficient clones, respectively (*SI Appendix*, Fig. S2*A* and Dataset S1). Although the average number of SNVs per cell was slightly lower in Polβ-deficient clones (62 ± 14) compared to wild-type clones (76 ± 29), the difference was not statistically significant (Fig. 1*B*). An alternative pathway, such as Polβ-independent long-patch BER (PCNA, FEN1, Polδ/ε, and Polλ), may be sufficient to suppress the accumulation of somatic SNVs, maintaining normal levels in response to stochastic DNA damage in developing neurons (13, 37).

### Loss of Polβ Frequently Induces Indel Mutations in Neurons.

The number of indels, particularly insertions, was higher in wild-type (123 ± 14/cell; range: 96 to 142) and Emx1-Cre/Polβ^fl/fl^ (111 ± 24/cell; range: 93 to 149) neurons than in adult HSCs (58 ± 12/cell) (Fig. 1*C*, *SI Appendix*, Fig. S2 *B* and *C*, and Dataset S2) (33). To investigate the mechanism of indel formation, sites were classified as nonrepeat, tandem repeat, and homopolymer. The most pronounced differences between wild-type and Polβ-deficient cells were found in nonrepeat sequences (Fig. 1 *D* and *E*). Insertions and deletions in nonrepeat sequences increased fourfold and 2.5-fold, respectively, in Emx1-Cre/Polβ^fl/fl^ cells (insertions: 7.3 ± 3.2/cell; deletions: 6.8 ± 2.9/cell) compared to wild-type cells (insertions: 1.7 ± 1.1/cell; deletions: 2.7 ± 1.1/cell). Polβ-deficient neurons frequently exhibited insertions ≥4 bp (Fig. 1*F*). The average insertion size was significantly larger in Polβ-deficient neurons (7.3 ± 3.7 bp) than in wild-type neurons (4.2 ± 3.5 bp, *P* = 0.015), whereas deletion sizes were comparable (wild-type: 4.9 ± 5.4 bp, Polβ-deficient: 5.1 ± 5.1 bp) (Fig. 1*G*). In contrast, adult HSCs displayed very few indels in nonrepeat sequences (Fig. 1*E*) (33). X-ray-induced DSBs efficiently promote deletions but not insertions (Fig. 1*E*) (33). These findings suggest that, in addition to error-prone DSB repair (38), Polβ mediates an insertion-prone repair mechanism in developing neurons.

The majority of indels occurred within homonucleotide runs and tandem repeat sequences (*SI Appendix*, Fig. S2 *D* and *E*). Although HSCs typically exhibit a similar number of insertions and deletions (33), cortical neurons exhibited a higher number of insertions than deletions in repeat sequences (*SI Appendix*, Fig. S2*E*). Notably, the number of insertions, but not deletions, within tandem repeat sequences per clone significantly decreased in a subset of Polβ-deficient cells (Fig. 1*H*). Indels in homopolymers were also comparable between wild-type and Polβ-deficient cells (*SI Appendix*, Fig. S2*F*). Thus, Polβ may selectively promote the expansion of tandem repeat sequences, though no significant multiunit of repeat expansion was observed. BER has been implicated in trinucleotide repeat expansions associated with oxidative damage in somatic cells (39, 40). However, the specific DNA polymerases responsible for this process in vivo remain unknown. In neurons, Polβ may play a role in this process.

### Absence of Polβ Induces Indel Mutations at CpG Sites.

**Fig. 2.**
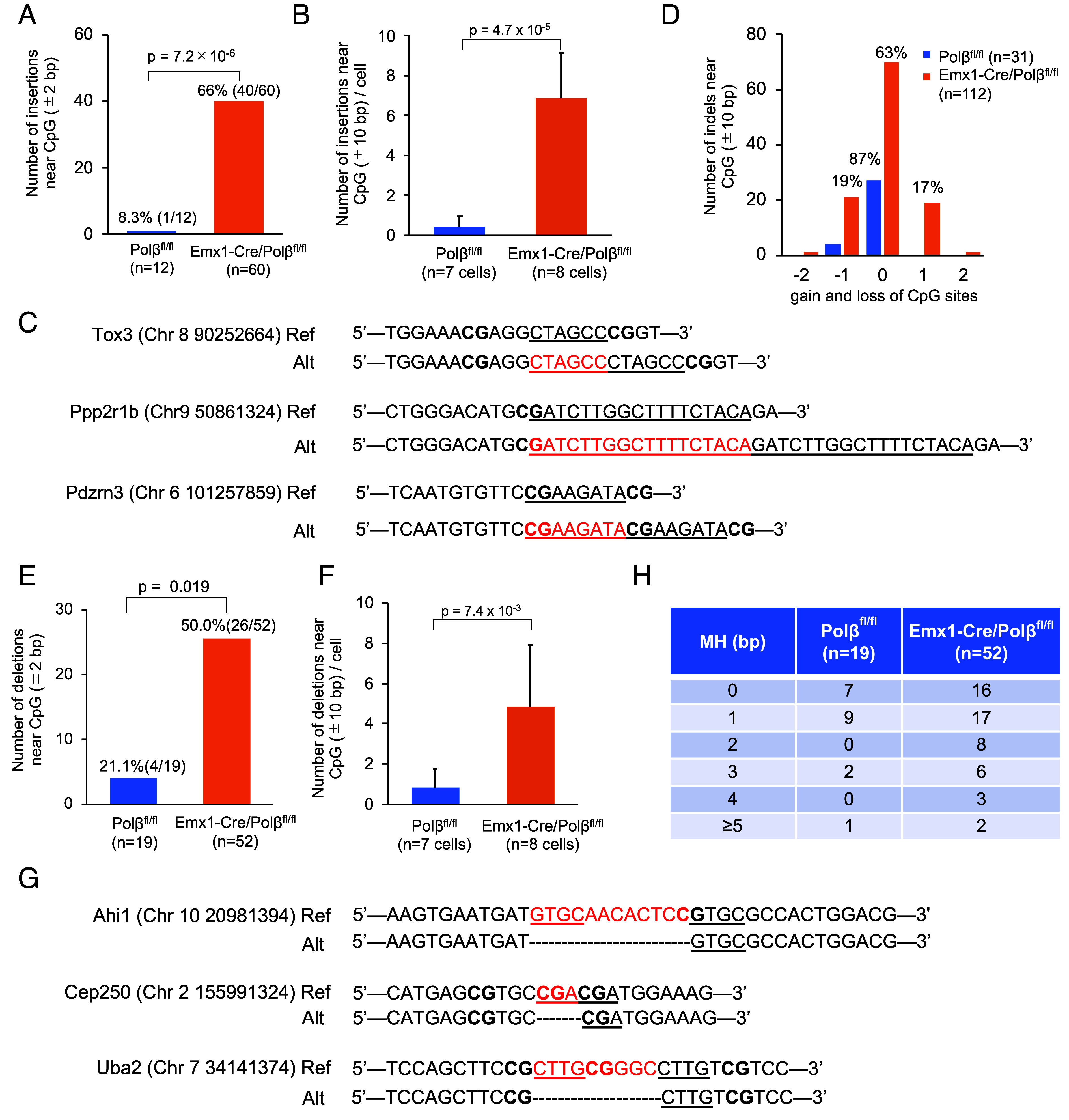
Loss of Polβ increases indel frequency in nonrepeat sequences rather than repeat sequences. (*A*) Total number of insertions with CpG sites within ±2 bp of the insertion sites in nonrepeat sequences of Emx1-Cre/Polβ^fl/fl^ and Polβ^fl/fl^ ntES cells. (*B*) Mean number of insertions near CpG sites (within ±10 bp) in Emx1-Cre/Polβ^fl/fl^ and Polβ^fl/fl^ ntES cells. (*C*) Examples of insertions in the coding region or 5′ UTR of Emx1-Cre/Polβ^fl/fl^ ntES cells. *Upper* and *Lower* rows show the reference sequence and mutated sequence, respectively. Bold text indicates CpG sites, red characters indicate inserted sequences, and underlined text indicates duplicated sequences. (*D*) Number of CpG sites gained and lost in indels near CpG site (within ±10 bp) in Emx1-Cre/Polβ^fl/fl^ and Polβ^fl/fl^ ntES cells. The numbers above the bar indicate percentages. (*E*) Total number of deletions with CpG sites within ±2 bp of the deletion sites in nonrepeat sequences of Emx1-Cre/Polβ^fl/fl^ and Polβ^fl/fl^ ntES cells. (*F*) Mean number of deletions near CpG sites (within ±10 bp) in Emx1-Cre/Polβ^fl/fl^ and Polβ^fl/fl^ ntES cells. (*G*) Examples of deletions in the coding region of Emx1-Cre/Polβ^fl/fl^ ntES cells. *Upper* and *Lower* rows show the reference sequence and mutated sequence, respectively. Bold text indicates CpG sites, red characters indicate deleted sequences, and underlined text represents microhomology observed in the junction sites. (*H*) Length distribution of microhomology observed at deletion junctions in nonrepeat sequences.

Forebrain development promotes cytosine demethylation at CpG dinucleotide sites (41, 42). To determine whether the loss of Polβ affects genome stability at CpG sites during active DNA demethylation, we analyzed CpG site frequency within ±100 bp of insertion sites. CpG sites were enriched at the insertion sites in Polβ-deficient cells, whereas no such accumulation was observed in wild-type cells (*SI Appendix*, Fig. S3*A*). CpG sites were significantly more frequent at the insertion sites (±2 bp) in Polβ-deficient cells (40/60) than in wild-type cells (1/12) (Fig. 2*A*). The frequency of insertions with CpG sites within ±10 bp was also significantly higher in Polβ-deficient cells (mean: 6.9/cell), showing a threefold to ninefold increase across biological replicates compared with wild-type cells (0.4/cell) (Fig. 2*B* and *SI Appendix*, Fig. S3 *B* and *C*). In some cases, multiple CpG sites were observed near the insertion sites (Fig. 2*C*). Furthermore, all insertions in the Polβ-deficient cells (60/60) and a substantial proportion in wild-type cells (9/12) exhibited sequence identity with subsequent downstream sequences, indicating duplications (Fig. 2*C*). Duplicated sequences in Polβ-deficient cells tended to have one additional CpG site (Fig. 2*D*).

Similar to insertions, CpG sites were significantly enriched at the deletion sites (±2 bp) in Polβ-deficient cells (26/52), in contrast to wild-type cells (4/19) (Fig. 2*E*). Polβ-deficient cells also exhibited a significantly higher frequency of deletions with CpG sites within ±10 bp of the deletion sites (mean: 4.8/cell), representing a ~fivefold increase over wild-type cells (0.9/cell, *P* = 7.4 × 10^−3^) (Fig. 2*F* and *SI Appendix*, Fig. S3 *D* and *E*). Loss of CpG sites by the deletions was increased in Polβ-deficient cells (Fig. 2*D*). These deletions were frequently associated with microhomology at the junction sequences in both Polβ-deficient (36/52) and wild-type (12/19) cells (Fig. 2 *G* and *H*). These findings suggest that Polβ deficiency leads to increased DSB formation at CpG sites. The accumulation of DSBs may overwhelm error-free repair pathways, thereby promoting error-prone repair through NHEJ and microhomology-mediated end-joining (MMEJ) (Fig. 5*F*) (38). Polβ may prevent mutagenesis at CpG sites during active DNA demethylation.

To determine whether these characteristic indels arise during neurogenesis, a process involving active DNA demethylation (42), we analyzed mutations in ntES cells derived from E18.5 Nex^Cre/+^/Polβ^fl/fl^ (Nex-Cre/Polβ^fl/fl^) neuron nuclei. In previous study, we reported that Nex-Cre/ Polβ^fl/fl^ neurons exhibit Polβ loss after the final mitosis but do not significantly accumulate DSBs in the developing cortex, unlike Emx1-Cre/ Polβ^fl/fl^ neurons (*SI Appendix*, Fig. S4*A*) (18, 19). Our results showed that the number of indels at CpG sites in these Polβ-deleted clones (clones F1-4) was comparable to that in wild-type clones (clone E1-4; *SI Appendix*, Fig. S4 *B*–*F* and Dataset S3). However, clone F2 cells exhibited a slight increase in insertions at CpG sites, similar to clones derived from Emx1-Cre/Polβ^fl/fl^ neuron nuclei (*SI Appendix*, Fig. S4*C*). In this case, Cre expression may have been induced earlier than basal Nex promoter activity, disrupting the Polβ gene in neural progenitors. These findings suggest that Polβ loss contributes to indels at CpG sites during neurogenesis. Furthermore, this experiment also indicates that the observed results are not due to the effects of Polβ loss in the donor nuclei on DNA demethylation during SCNT reprogramming or the subsequent culture of ntES cells (14, 43). Whether Polβ is involved in the process of DNA demethylation during SCNT reprogramming and ntES cell establishment remains unclear. Presumably, other DNA polymerases may compensate for the loss of Polβ in this process.

### Polβ Deficiency Alters Mutation Signatures.

**Fig. 3.**
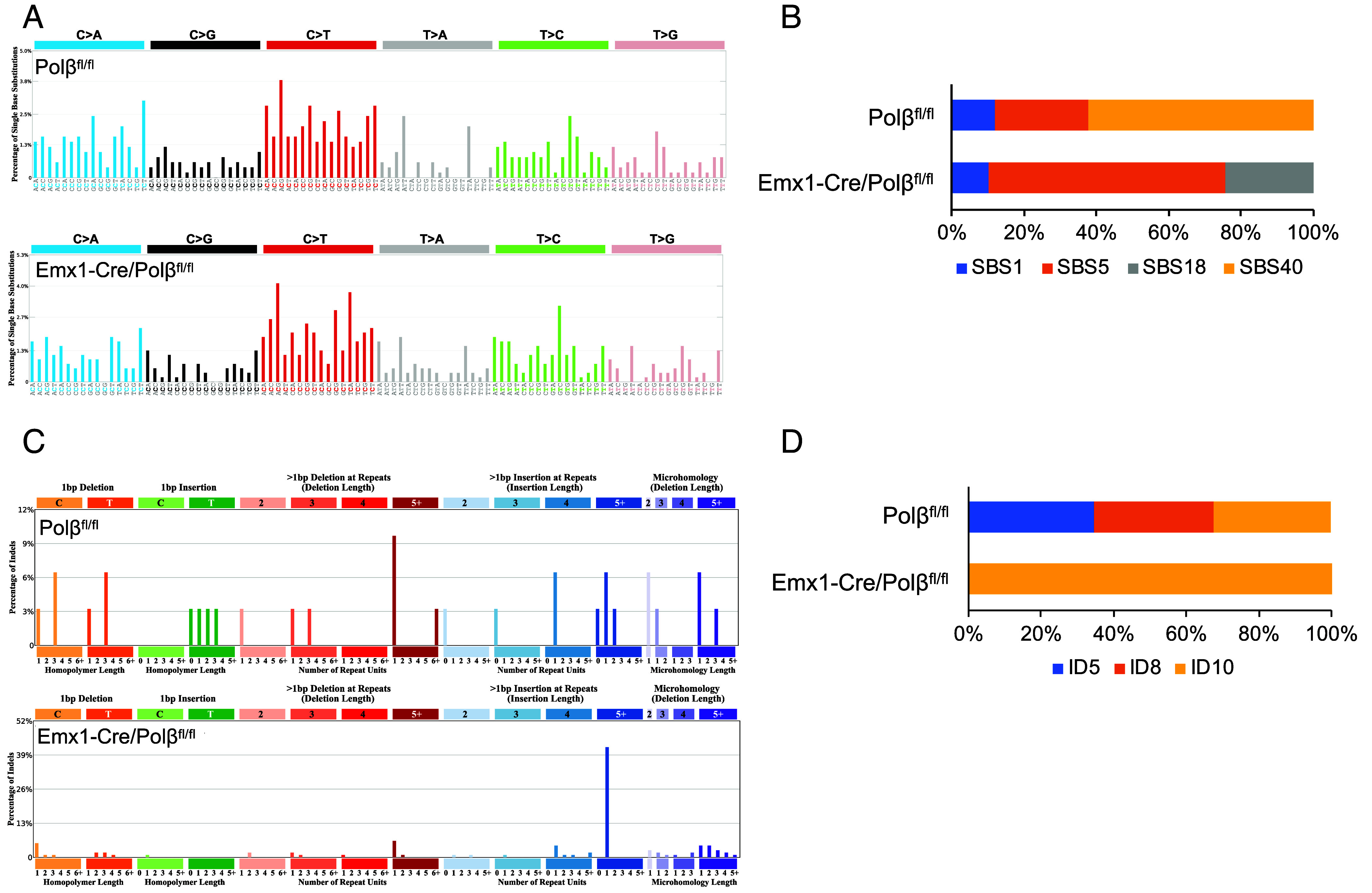
Somatic mutation signatures in cortical neurons. (*A*) Trinucleotide context of somatic substitutions in Emx1-Cre/Polβ^fl/fl^ (535 substitutions, n = 8 cells) and Polβ^fl/fl^ (494 substitutions, n = 7 cells) ntES cells. (*B*) Contribution of COSMIC SBS signatures in Emx1-Cre/Polβ^fl/fl^ and Polβ^fl/fl^ ntES cells. (*C*) Mutation spectrum of somatic indels in nonrepeat sequences of Emx1-Cre/Polβ^fl/fl^ and Polβ^fl/fl^ ntES cells. (*D*) Contribution of COSMIC ID signatures in Emx1-Cre/Polβ^fl/fl^ and Polβ^fl/fl^ ntES cells.

Signature analysis using the COSMIC database (44) identified a distinctive feature of indels Polβ-deficient cells: insertions exceeding 5 bp in nonrepeat sequences, predominantly duplications (Fig. 3*C*). Control Polβ^fl/fl^ cells exhibited a signature with 35% ID5, 33% ID8, and 32% ID10 (Fig. 3*D* and *SI Appendix*, Fig. S5*A*). In Polβ-deficient cells, the signature is predominantly associated with ID10. ID10, characterized by duplications over 5 bp, has been identified in certain cancers with an unknown mechanism. This finding suggests that Polβ deficiency preferentially induces ID10, which may provide insight into a novel mechanism. ID5 and ID8, previously reported in human neurons, are known to accumulate in a clock-like manner (3, 4). Meanwhile, single base substitution (SBS) analysis indicated a higher prevalence of C > T mutations, likely resulting from methylated cytosine deamination, in both wild-type and Polβ-deficient cells (Fig. 3*A*). This pattern is consistent with previous observations in human neurons and mouse HSCs but diverges from the substitution signature identified in mouse adult tail fibroblasts using ntES cell lines (4, 31, 33). The mutational profile in Polβ-deficient cells was distinct from that of wild-type cells, primarily characterized by SBS40 and SBS18 (Fig. 3*B*). Importantly, SBS40 has been linked to SV formation (45) and increases in a dose-dependent manner upon X-ray irradiation in HSCs (33). SBS18, commonly observed in neuroblastoma (45, 46), is particularly relevant given that Polβ deficiency promotes medulloblastoma in p53 null mice (47). These findings suggest that Polβ loss alters both ID and SBS signatures in cortical neurons, likely due to the engagement of alternative repair pathways, such as NHEJ, MMEJ, and long-patch BER (13, 37, 38).

### Loss of Polβ Induces SVs but Not MEIs.

**Fig. 4.**
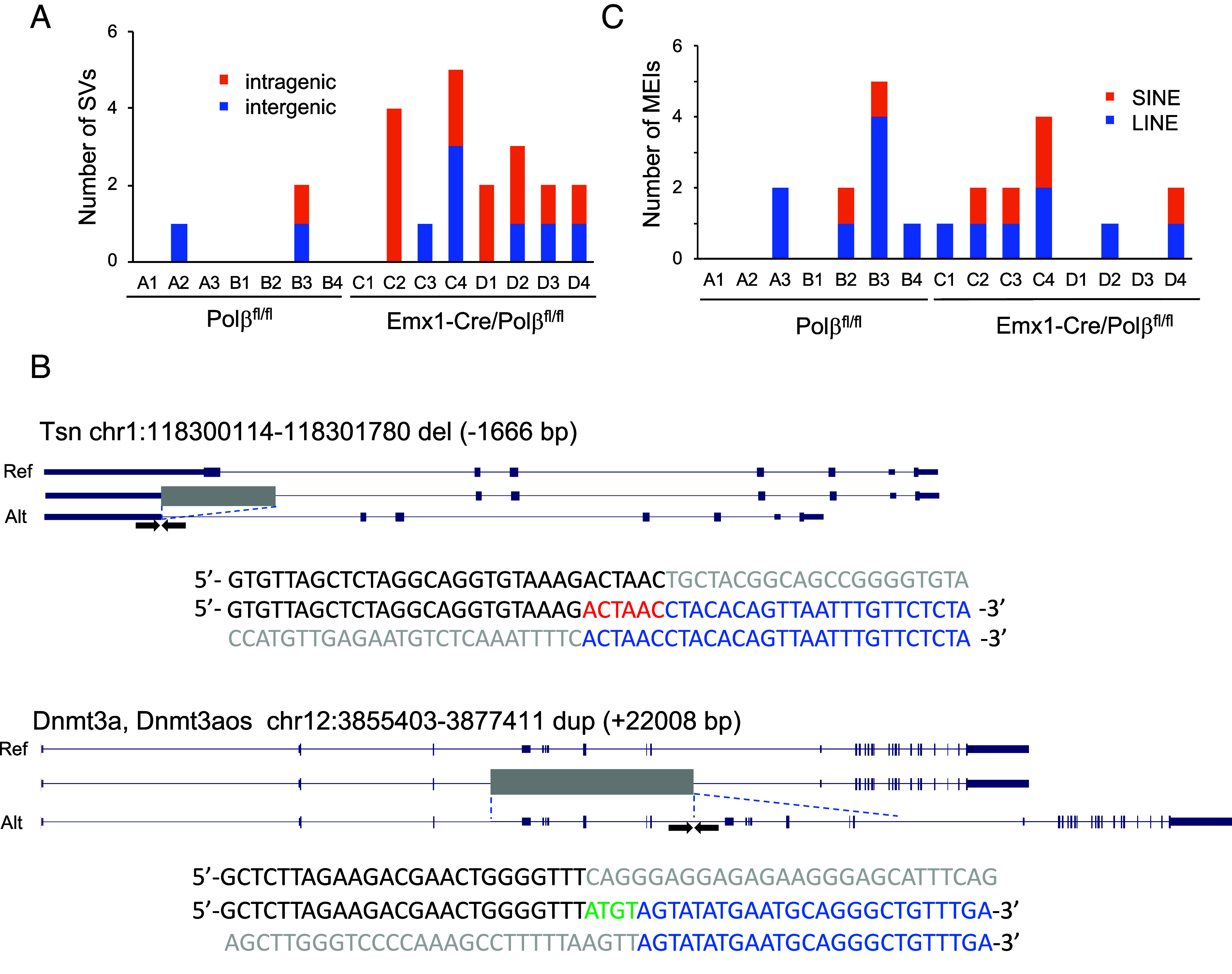
Loss of Polβ increases the number of SVs but not MEIs. (*A*) Number of somatic SVs in each Emx1-Cre/Polβ^fl/fl^ and Polβ^fl/fl^ ntES cell. (*B*) Examples of SV structures detected in the intragenic regions of Emx1-Cre/Polβ^fl/fl^ ntES cells. The *Top*, *Middle*, and *Bottom* rows indicate the reference gene structure, the position of the deletion or duplication regions (gray), and the altered gene structure, respectively. Black and blue characters show the 5′ and 3′ junction sequences of SVs, respectively, flanked by primer sets (arrows). Microhomology, insertion, and deletion sequences are indicated in red, green, and gray, respectively. (*C*) Number of somatic LINE and SINE insertions in each Emx1-Cre/Polβ^fl/fl^ and Polβ^fl/fl^ ntES cell.

Increased DSBs are known to induce SV formation (48). Additionally, DNA demethylation regulates the induction of mobile element insertions (MEIs), such as long interspersed elements (LINEs) and short interspersed elements (SINEs), which contribute to somatic mutations linked to psychiatric disorders (2, 6). To investigate this further, we analyzed SVs and MEIs in ntES cells using multiple detection tools (SvABA, Manta, and RUFUS) (49–51). We identified 19 SVs, comprising 16 large deletions and three duplications, in Polβ-deficient cells (size 50 bp to 93 kbp, 2.3 ± 1.4 per cell). This represents an ~fivefold increase over wild-type cells, in which only three variants (size: 5 kbp to 40 kbp, two deletions and one duplication) were detected (0.43 ± 0.79 per cell) (Fig. 4*A* and *SI Appendix*, Fig. S6). This finding suggests a critical role of Polβ in the suppression of SV formation. The junction sequences of these SVs exhibited microhomology, with no significant enrichment of CpG sites (Fig. 4*B*). More than half of SVs were located in intragenic regions, potentially affecting regulatory elements and coding sequences of genes such as *Tsn* and *Dnmt3a* (Fig. 4 *A* and *B*). In contrast, the frequency of MEIs did not significantly differ between Polβ-deficient cells (1.5 ± 1.4 per cell; range: 0 to 4) and wild-type cells (1.4 ± 1.4 per cell; range: 0 to 5) (Fig. 4*C* and *SI Appendix*, Fig. S7). Thus, Polβ deficiency promotes SV formation but does not significantly impact MEI frequency, likely through DSB-induced repair via NHEJ or MMEJ in cortical neurons.

### Polβ-Mediated Indels Affect Both Regulatory and Coding Regions in Neuronal Genes.

**Fig. 5.**
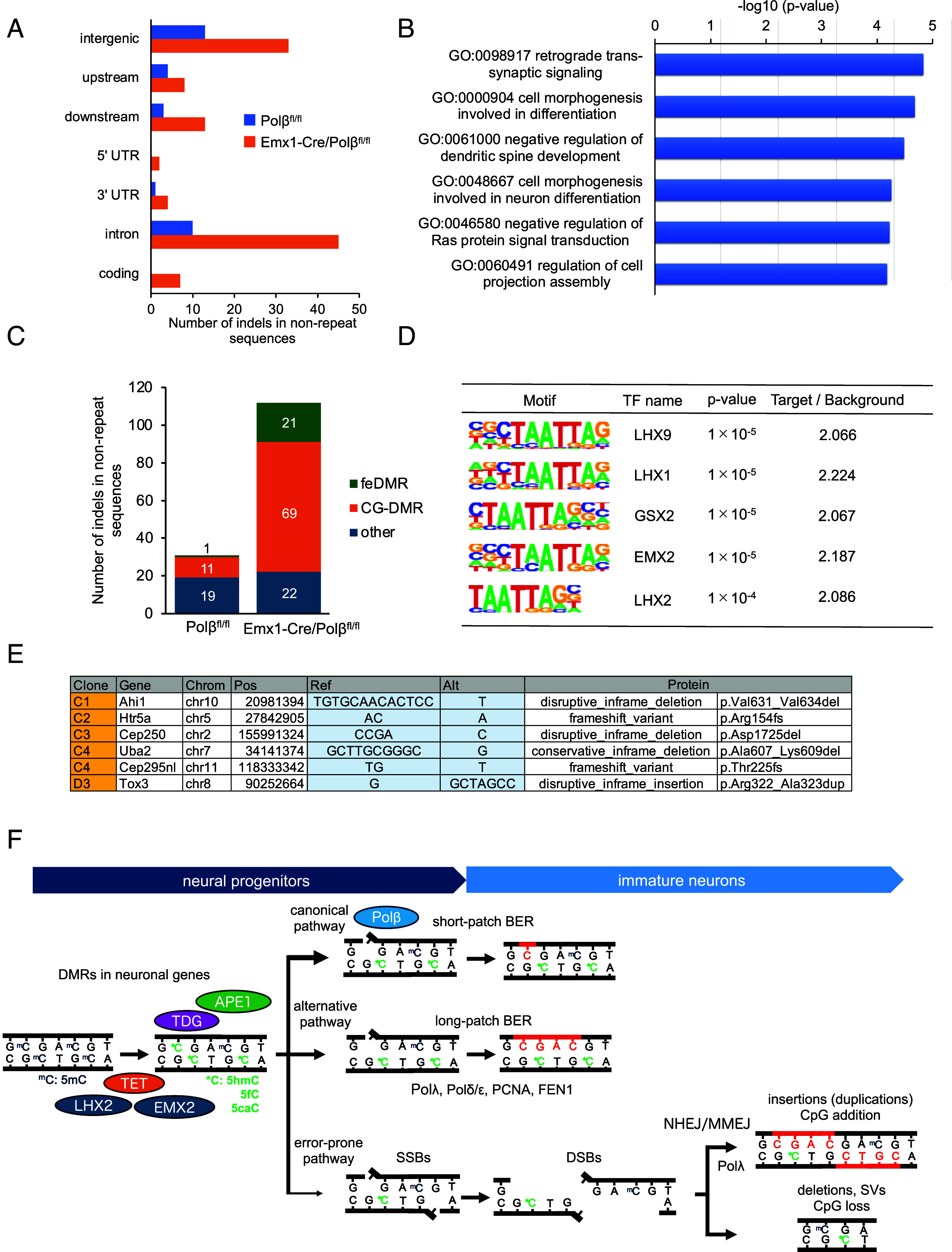
Somatic indels in nonrepeat sequences affect neuronal genes. (*A*) Genomic distribution of indel sites in Emx1-Cre/Polβ^fl/fl^ and Polβ^fl/fl^ ntES cells. (*B*) Top six Gene Ontology biological processes associated with genes containing intragenic mutations in nonrepeat sequences in Emx1-Cre/Polβ^fl/fl^ and Polβ^fl/fl^ ntES cells. (*C*) Number of indels near CpG sites located in feDMRs and CG-DMRs in Emx1-Cre/Polβ^fl/fl^ and Polβ^fl/fl^ ntES cells. (*D*) Top five transcription factor binding motifs enriched within ±200 bp of indel sites. (*E*) Indels detected within the coding regions in Emx1-Cre/Polβ^fl/fl^ ntES cells. (*F*) Schematic model illustrating Polβ-mediated indel and SV formation driven by active DNA demethylation during cortical neurogenesis.

In human neurons, mutations are frequently observed in actively transcribed neuronal genes with open chromatin (3, 52, 53). We analyzed the functional impact of somatic indels in nonrepeat sequences. In Polβ-deficient cells, indels were enriched in putative regulatory regions within the gene bodies, particularly in the 5′ and 3′ untranslated regions (UTRs) and introns (Fig. 5*A*). Moreover, gene ontology analysis showed enrichment of genes involved in synaptic signaling and neuronal morphogenesis (Fig. 5*B*). We further analyzed the positional relationship between indels and differentially methylated CpG regions (CG-DMRs) in developing mouse fetus using previously reported data (54). Notably, indel sites were predominantly localized within CG-DMRs, with a subset found in fetal enhancer-linked CG-DMRs (feDMRs), which are associated with putative transcriptional regulatory regions during forebrain development (Fig. 5*C*) (54). To identify transcription factors that bind to these regions, we performed motif analysis using HOMER (55). This analysis revealed an enrichment of DNA binding sites for key cortical development factors, such as LHX2 and EMX2 (56) (Fig. 5*D*). This suggests that the indels may influence neuronal gene expression during cortical development. Furthermore, Polβ-deficient cells exhibited one insertion and five deletions in coding regions (5/8 cells), whereas no such events were detected in wild-type cells (Fig. 5*E*). These indels caused in-frame alterations, resulting in amino acid insertions or deletions, as well as frameshift mutations. Of particular interest, they affected neuronal genes, including *Ahi1* (implicated in Joubert syndrome), *Tox3* (a calcium-dependent transactivator), and *Htr5a* (a serotonin receptor), all of which are implicated in neuronal function and psychiatric disorders (57–59). Thus, Polβ-mediated somatic indels occurring during cortical development impact multiple neuronal genes through both regulatory and coding alterations, which may affect individual neuronal function.

## Discussion

In this study, we investigated somatic mutations in immature cortical neurons using SCNT and WGS. Our results reveal that Polβ deficiency in neural progenitor cells leads to a ~ninefold increase in indels at CpG dinucleotides and a ~fivefold rise in SVs. These indels were enriched in neuronal genes, with some leading to frameshift mutations or alterations of CpG sites in putative transcriptional regulatory regions. Our findings suggest that Polβ preferentially repairs DNA lesions at CpG sites generated by TET-mediated active DNA demethylation, thereby suppressing the mutagenesis that accompanies neuronal gene activation during cortical development (Fig. 5*F*).

While Polβ loss in neural progenitor cells has been linked to increased DSB formation and apoptosis during neurogenesis (19), the precise mutation spectrum in neurons remained unclear. We reveal that CpG-specific indels are a hallmark of Polβ-deficient immature neurons, suggesting that these mutations predominantly arise during neurogenesis. This finding supports the hypothesis that TET-dependent active CpG demethylation contributes to mutagenesis via its interaction with BER, a process previously uncharacterized in vivo (11, 14, 60). During differentiation, active DNA demethylation may occur simultaneously at multiple CpG sites within DMRs, requiring efficient repair. If the canonical Polβ-dependent BER pathway is impaired, an alternative pathway, long-patch BER, may be insufficient, leading to DSB formation (13, 23, 37, 61). In such cases, error-prone DSB repair pathways, such as NHEJ and MMEJ, may introduce indels (12, 38). Our data suggest that the predominant repair mechanism for active DNA demethylation-associated lesions in neural progenitor cells relies on Polβ.

A striking feature of the observed indels is the frequent occurrence of duplications in addition to deletions. This contrasts with random DSBs induced by X-ray irradiation, which primarily result in deletions (33). Interestingly, a similar phenomenon is observed in the AID-dependent immunoglobulin hypermutation process, where Polλ promotes duplications in Polβ-deficient B cells (62). This raises the possibility that Polλ-dependent duplications also occur during neurogenesis, which warrants further investigation. Given the parallels between AID-mediated mutagenesis in B cells and active DNA demethylation during neurogenesis in Polβ-deficient cells, it is plausible that concurrent SSBs at adjacent cytosines leading to DSBs, coupled with Polλ-mediated NHEJ, contribute to duplications. Meanwhile, the frequent occurrence of deletions and SVs suggests that DSBs might arise simultaneously at multiple CpG sites within DMRs. Indeed, SVs were frequently observed within genes, and the nature of mutations may depend on the status of DSB termini and the activity of end-processing factors (38).

The accumulation of indels at CpG sites within DMRs during development suggests that genomic instability in regulatory regions of cell type–specific genes is selectively and transiently elevated. Variations in CpG sites within transcriptional regulatory regions can directly impact gene expression, and such mutations may contribute to neurodevelopmental disorders (63). Supporting this notion, WGS studies of postmortem schizophrenia brains have reported increased CpG > GpG transversions at transcription factor binding sites, possibly linked to active DNA demethylation (64). Consistent with this, we observed a slight increase in CpG > GpG transversions in Polβ-deficient cells, although the trend was not statistically significant (*P* = 0.069, *SI Appendix*, Fig. S5*B*). Further analysis with a larger sample size is needed to confirm this trend; however, our findings provide insights into the molecular mechanisms underlying these mutations.

Our results further suggest that Polβ may be linked to an increased mutation burden during human cortical development, a phenomenon known as brain mosaicism. Active DNA demethylation at neuronal enhancer sites induces SSBs in cultured human neurons, suggesting a mechanism through which CpG-site instability may arise (65–67). We analyzed previously published single-cell WGS data from postmortem human brain samples and found that somatic indels occurring near CpG sites within ±10 bp were most frequent in infant neurons (28.5%, 6/21 indels) compared to adolescence (11.2%, 25/222) and adulthood (15.1%, 348/2,538) (53). Moreover, most of these indels (5/6) in infant neurons were localized in intron regions, suggesting that they may have arisen via active DNA demethylation during neuronal differentiation. While no direct link between Polβ and brain disorders has been established, these findings suggest that active DNA demethylation-induced indel formation at CpG sites may be a conserved mutagenic mechanism in humans.

SCNT-based approaches are effective for analyzing de novo mutations in somatic cells (31, 32). However, when investigating Polβ-deficient cells, global DNA demethylation during SCNT reprogramming must be considered (14, 43). To determine whether Polβ is required for reprogramming, we used Polβ-deficient donor nuclei from Nex-Cre/Polβ^fl/fl^ neurons with wild-type enucleated recipient cells. If Polβ were essential, we would expect increased indels at CpG sites in Polβ-deficient ntES cells, independent of Cre-mediated deletion timing. However, indel levels were comparable to those in wild-type cells, suggesting that Polβ is not critical for SCNT reprogramming (*SI Appendix*, Fig. S4). Alternatively, maternally supplied Polβ may be sufficient. Thus, the detected CpG site indels in ntES cells derived from Emx1-Cre/Polβ^fl/fl^ neurons likely reflect mutagenesis occurring in neural progenitor cells during neurogenesis rather than during reprogramming.

Detecting rare indels in homopolymers and tandem repeats remains challenging (3, 68). In this study, we found that Polβ deficiency specifically reduced insertions in tandem repeats but did not affect deletions (Fig. 1*H*). Moreover, homopolymers remained unaffected (*SI Appendix*, Fig. S2*F*). Given that somatic tandem repeat expansion under oxidative conditions requires mismatch repair and BER, Polβ may play a role in this process (40, 69). Somatic expansion of tandem repeats is associated with age-related neurodegeneration and psychiatric disorders (39, 40, 70). The impact of Polβ loss on tandem repeat stability underscores the need for long-term studies to clarify its function in neurons throughout life.

Our findings reveal that Polβ-mediated repair of active DNA demethylation is essential for maintaining genome stability in developing neurons, highlighting its potential involvement in neurodevelopmental disorders. Loss of Polβ triggers a ~ninefold increase in indels at actively demethylated CpG sites, which alter coding or regulatory sequences in neuronal genes. By demonstrating that active DNA demethylation can become mutagenic unless swiftly repaired, our work provides a mechanism that links epigenetic gene activation to the somatic mutations detected in various brain disorders. A deeper understanding of the interplay between epigenome regulation and DNA repair across development and aging is critical for identifying risk factors and therapeutic targets (52, 53, 70, 71).

## Materials and Methods

### Animals.

All experiments were conducted in accordance with the guidelines for the care and use of laboratory animals of Osaka University and University of Yamanashi. Emx1-Cre/Polβ^fl/fl^ and Nex-Cre/Polβ^fl/fl^ mice were generated as previously described (19). B6D2F1 mice (Japan SLC) were used as oocyte donors. Both male and female mice were included in all experiments. Noon of the day on which the vaginal plug was detected was designated as embryonic day 0.5 (E0.5).

### Preparation of Dissociated Cortical Neuronal Cells.

Pregnant mice were deeply anesthetized with isoflurane, and neocortices were dissected from E18.5 embryos in ice-cold Hanks’ balanced salt solution. The neocortices were minced using fine scissors in phosphate-buffered saline (PBS; pH 7.4), and the tissue fragments were incubated in PBS containing 0.125% trypsin and 0.02% ethylenediaminetetraacetic acid (EDTA) at 37 °C for 5 min. To stop trypsin activity, DMEM/F-12 culture medium supplemented with 10% fetal bovine serum (FBS) was added. The digested tissue was then gently triturated using a fire-polished Pasteur pipette. After centrifugation, the cells were resuspended in CellBanker 1Plus freezing medium (Takara Bio) and stored at −80 °C until nuclear transfer.

### Nuclear Transfer and Establishment of ntES Cell Lines.

Nuclear transfer was performed as previously described (29). Neurons dissociated from E18.5 Emx1-Cre/Polβ^fl/fl^, Nex-Cre/Polβ^fl/fl^, and Polβ^fl/fl^ cortices were used as nuclear donors. Following nuclear transfer, reconstructed oocytes were activated using 5 mM SrCl_2_ in Ca-free CZB medium in the presence of 5 μM latrunculin A and 50 nM trichostatin A for 9 h. After three washes in CZB, cloned embryos were cultured for 4 d in the same medium, and morula- or blastocyst-stage embryos were used to establish ntES cell lines as previously described (30).

### WGS Analysis.

High-molecular-weight genomic DNA was extracted from ntES cells using the DNeasy Blood & Tissue Kit (Qiagen). Extracted DNA was used to prepare paired-end libraries according to the Illumina Sample Preparation Kit user manual, without PCR amplification. The libraries were sequenced using the Illumina NovaSeq platform with 150 bp paired-end reads. The samples had an average total read depth of 30× per nucleotide. Sequence reads were mapped to a mouse reference genome (mm10) using the Burrows–Wheeler aligner with the maximal exact matches (BWA-MEM) algorithm.

### Mapping and Variant Calling.

We used BWA-MEM v0.7.17 with the “–M” option for Picard compatibility to map sequence reads to the mouse reference genome (UCSC mm10). Subsequently, we performed deduplication and base-quality recalibration using Picard v2.18.26. For SNV and small indel calling, to minimize false variant calls, we exclusively used only highly reliable (HR) reads meeting the following conditions were extracted: 1) properly mapped according to the aligner, 2) mapping with a quality score of ≥60 using SAMtools-1.9 (samtools view -q 60 -f 0 × 2 -F 0 × 500) (72), and 3) mapping to the reference without clipping. To ensure accurate identification and comparison of somatic mutations across all samples (31, 73), we defined effective whole-genome coverage (EWC) regions that satisfied the following criteria: 1) MQ60 read depths within 50 to 300% of the peak coverage for each chromosome, 2) a depth ratio of MQ60 reads to all mapped reads of ≥80% at each site, and 3) a minimum base quality of 20 according to SAMtools. These EWC regions were initially defined for each sample. Subsequently, the regions shared among all the samples were used for mutation analyses, covering 82.9% and 80.7% of diploid autosomes in Emx1-Cre/Polβ^fl/fl^ and Nex-Cre/Polβ^fl/fl^ mice, respectively (*SI Appendix*, Figs. S8 and S9). Genomic variants, compared with the mouse reference genome, were called using GATK v4.1.0.0 HaplotypeCaller (34). De novo mutation candidates were identified by filtering variants with the following criteria: a variant allele frequency >30% in a cell clone and <1% in sister cell clones. Validation of candidates involved visual inspection using Integrative Genomics Viewer (IGV) and verified variants were defined as de novo somatic mutations. Indel variants were divided into two groups: those occurring in repeat sequences (where the same motif appeared ≥5 times) and those in other nonrepeat sequences. ToppGene Suite (https://toppgene.cchmc.org) was employed to characterize biological processes associated with identified somatic SNVs and indels.

Mutation calling for SVs and MEIs was performed using SvABA v1.0.1, Manta v1.6.0, and RUFUS (49–51). In SvABA, variants were called based on the following inclusion criteria: 1) an allele depth (AD) > 5 in a clone and 2) an AD of 0 or 1 in all clones from the other clones. In Manta, the variants were called based on the following inclusion criteria: 1) a split reads for alternative allele (SRA) value ≥ 4 in a clone and 2) an SRA of 0 in all clones from the other mice. RUFUS, run with one clone as the subject and the others as controls, called variants based on the following criteria: 1) SVTYPE INFO in a clone, 2) other INFO with ALT_LEN ≥ 30 in a clone, or 3) other INFO with REF_LEN ≥ 30 in a clone. All identified candidate variants in neuron-derived clones were manually validated using IGV, with the analysis not limited to EWC regions.

### Mutation Signatures.

Mutational signature analysis and visualization of single-base substitutions within the EWC regions were performed using SigProfilerMatrixGenerator v1.2.2 (74) and SigProfilerExtractor v1.1.4, with the parameters “cosmic_version = 3.2,” “reference_genome = mm10,” “opportunity_genome = mm10,” and “nmf_replicates = 100” (44). Mutational signatures for each sample were categorized through hierarchical clustering using Ward’s minimum variance method (Ward. D2) employing the heatmap.2 function from the “gplots” package in R. Motif analysis for indel sites was performed with HOMER with its database of known transcription factor motifs (55). The enrichment of feDMRs and CG-DMRs at indel sites was analyzed using DeepTools and ENCODE epigenome data: CG-DMRs (thumper-e1.ENCODE_mouse_tissues.DMR_CG_ENCODE_4DMS) and feDMR (thumper-e1.ENCODE_mouse_tissues.feDMR_FB). These datasets were obtained from the ENCODE Mouse Fetal Development project (http://neomorph.salk.edu/ENCODE_mouse_fetal_development.html) (54, 75).

### Statistical Analysis.

For each experiment, the number of samples analyzed is provided. Welch’s *t* test was used to determine significant differences, and summary statistics are presented as means ± SD. All data analyses were performed using Microsoft Excel and GraphPad Prism.

### Declaration of Generative AI and AI-Assisted Technologies in the Writing Process.

During the preparation of this manuscript, the authors used ChatGPT4 and DeepL exclusively for grammatical error correction and stylistic changes. The authors thoroughly reviewed and edited the content following the use of these tools and take full responsibility for the final content of the publication.

## Supplementary Material

Appendix 01 (PDF)

Dataset S01 (XLSX)

Dataset S02 (XLSX)

Dataset S03 (XLSX)

## Data Availability

Raw sequencing data have been deposited in the DNA Data Bank of Japan Sequence Read Archive (DDBJ‐SRA) under accession numbers (BioProject: PRJDB20767) (76). Data are publicly available at https://ddbj.nig.ac.jp/sra.
