# Supplementary material for: DNA polymerase β suppresses somatic indels at CpG dinucleotides in developing cortical neurons
Source: Proc Natl Acad Sci U S A. 2025 Aug 13;122(33):e2506846122. doi: 10.1073/pnas.2506846122 (PMC12377747; doi:10.1073/pnas.2506846122)
Supplement: Supplementary file 1 — Appendix 01 (PDF) [file pnas.2506846122.sapp.pdf]

PNAS

Supporting Information for

DNA polymerase β suppresses somatic indels at CpG dinucleotides in developing cortical neurons

Noriyuki Sugo, Arikuni Uchimura, Risa Matsumoto, Hiro Nakayama, Shota Fujimoto, Saya Mizuno, Mayumi Higuchi, Masaaki Toshishige, Yasunari Satoh, Sayaka Wakayama, Teruhiko Wakayama, Takeshi Yagi

Noriyuki Sugo
Email: sugo@fbs.osaka-u.ac.jp

This PDF file includes:

Figures S1 to S9

Other supporting materials for this manuscript include the following:

Datasets S1 to S3

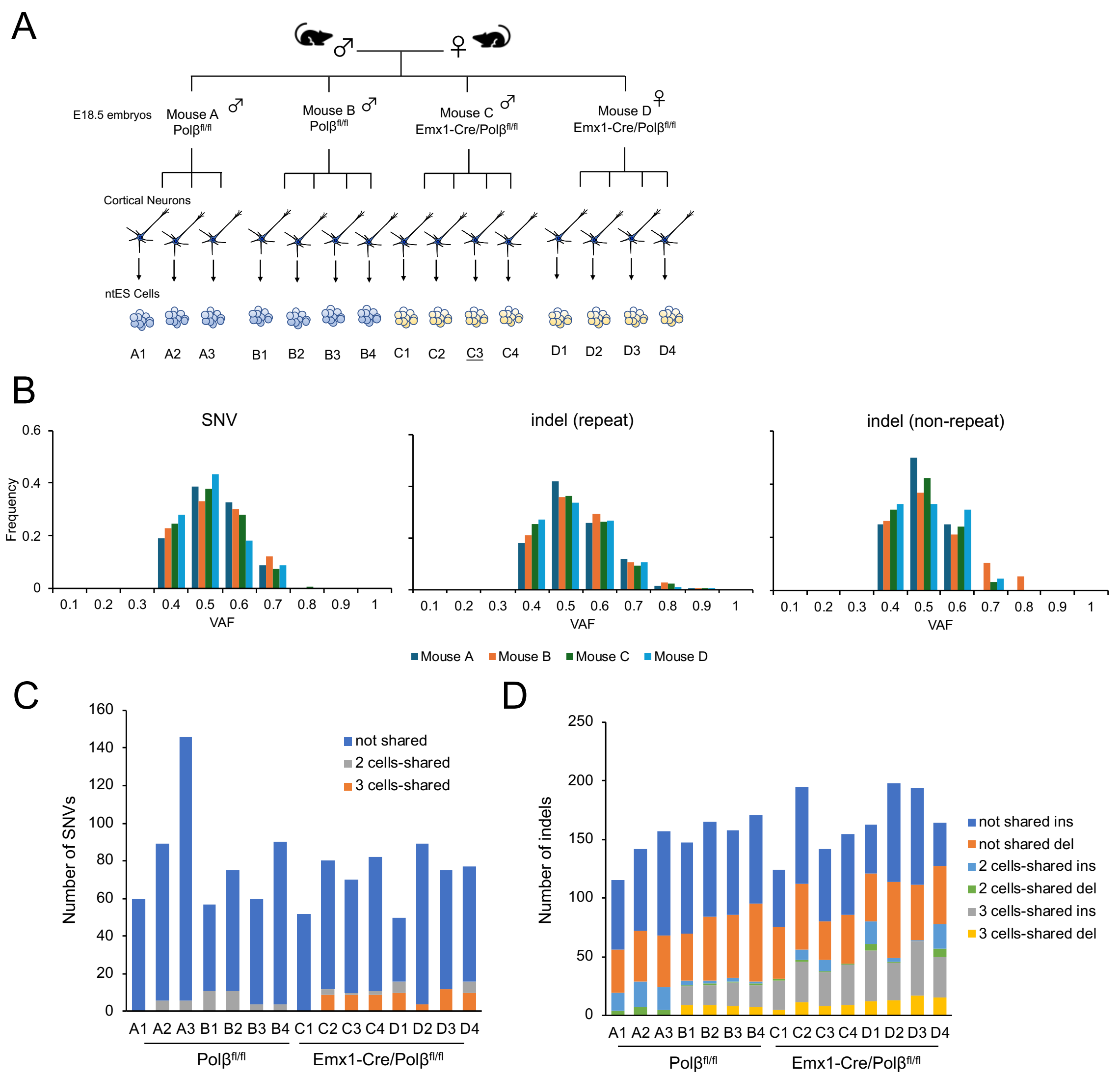

Fig. S1. (**A**) Schematic representation of E18.5 littermate embryos (Emx1-Cre/Polβ$^{fl/fl}$ and Polβ$^{fl/fl}$) and the corresponding ntES cell lines derived from cortical neurons in each mouse. (**B**) Distribution of VAF in SNVs, repeat indels, non-repeat indels identified in each ntES cell derived from mice A, B, C, and D. (**C**) Number of somatic SNVs in each ntES cell line established from cloned mouse embryos derived from Emx1-Cre/Polβ$^{fl/fl}$ and Polβ$^{fl/fl}$ cortical neuron nuclei, including the composition of mutations shared or unshared among ntES clones from the same cortex. (**D**) Number of somatic indels in each ntES cell line of Emx1-Cre/Polβ$^{fl/fl}$ and Polβ$^{fl/fl}$ mice, including shared or unshared mutations.

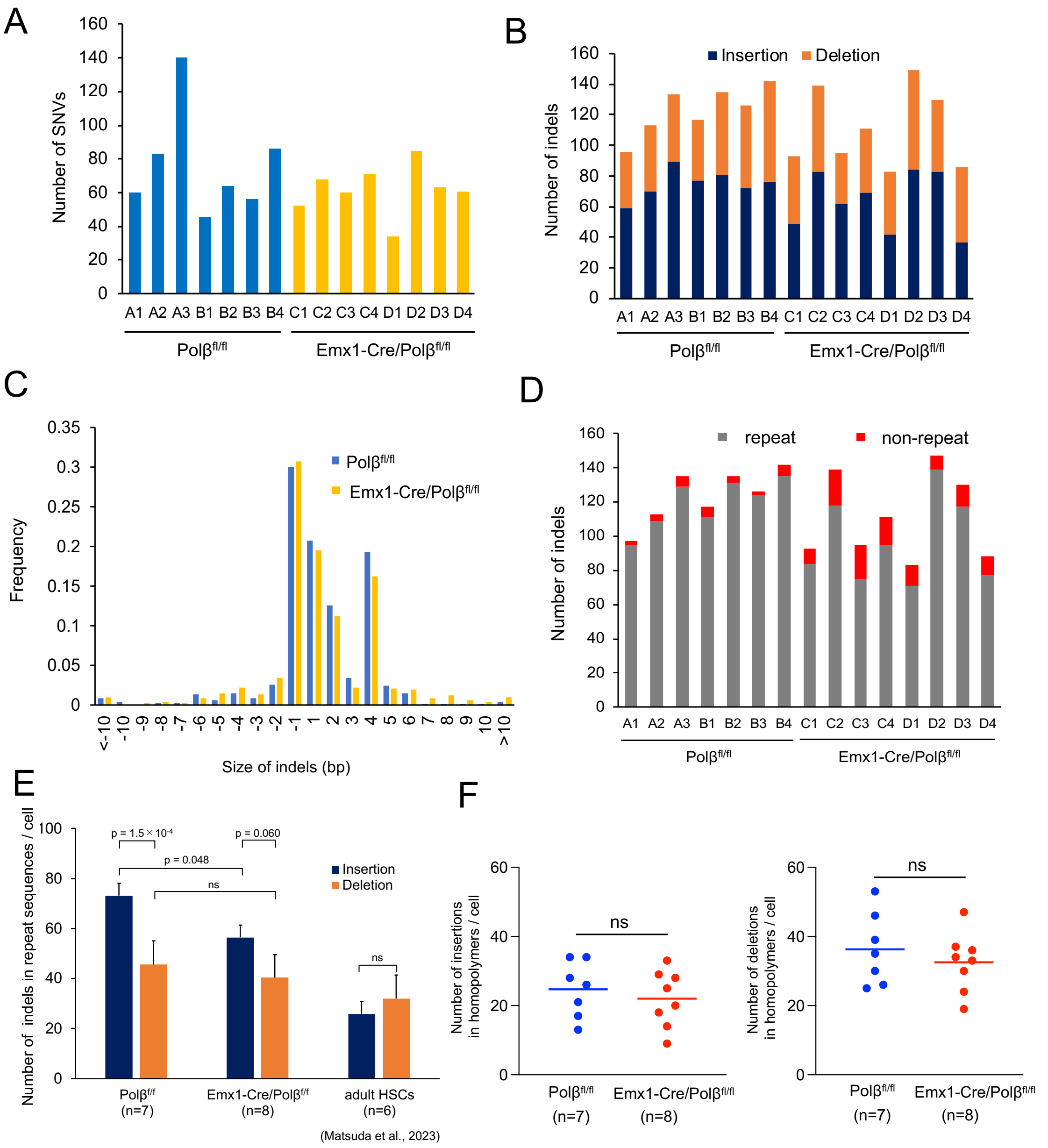

Fig. S2. (**A**) Number of somatic SNVs in each ntES cell line from Emx1-Cre/Polβ$^{fl/fl}$ and Polβ$^{fl/fl}$ cortical neuron nuclei. (**B**) Number of somatic insertions and deletions in each Emx1-Cre/Polβ$^{fl/fl}$ and Polβ$^{fl/fl}$ ntES cell line. (**C**) Distribution of indel sizes in Emx1-Cre/Polβ$^{fl/fl}$ and Polβ$^{fl/fl}$ ntES cells. (**D**) Number of indels in repeat and non-repeat sequences in each Emx1-Cre/Polβ$^{fl/fl}$ and Polβ$^{fl/fl}$ ntES cell. (**E**) Mean number of insertions and deletions in repeat sequences of Emx1-Cre/Polβ$^{fl/fl}$ and Polβ$^{fl/fl}$ ntES cells as well as in adult HSCs. (**F**) Number of indels in homopolymer sequences in each Emx1-Cre/Polβ$^{fl/fl}$ and Polβ$^{fl/fl}$ ntES cell.

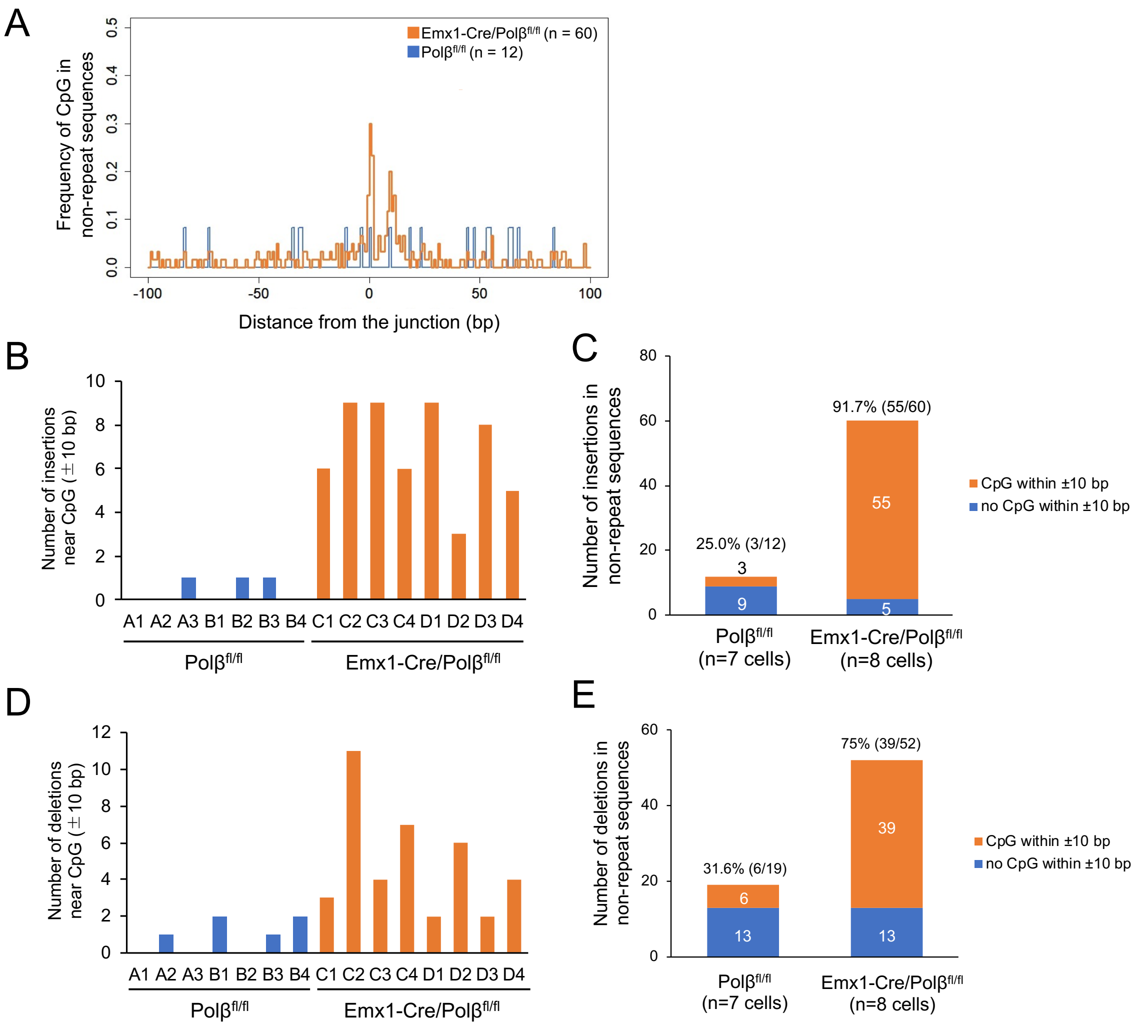

Fig. S3. (**A**) Frequency of CpG sites within ±100 bp from insertion junctions in Emx1-Cre/Polβ$^{fl/fl}$ and Polβ$^{fl/fl}$ ntES cells. (**B**) Number of insertions with CpG sites within ±10 bp of insertion sites in each Emx1-Cre/Polβ$^{fl/fl}$ and Polβ$^{fl/fl}$ ntES cell. (**C**) Total number of insertions in non-repeat sequences in Emx1-Cre/Polβ$^{fl/fl}$ and Polβ$^{fl/fl}$ ntES cells, categorized by the presence or absence of CpG sites within ±10 bp. (**D**) Number of deletions with CpG sites within ±10 bp of deletion sites in each Emx1-Cre/Polβf $^{fl/fl}$ and Polβ$^{fl/fl}$ ntES cell. (**E**) Total number of deletions in non-repeat sequences in Emx1-Cre/Polβ$^{fl/fl}$ and Polβ$^{fl/fl}$ ntES cells, categorized by the presence or absence of CpG sites within ±10 bp.

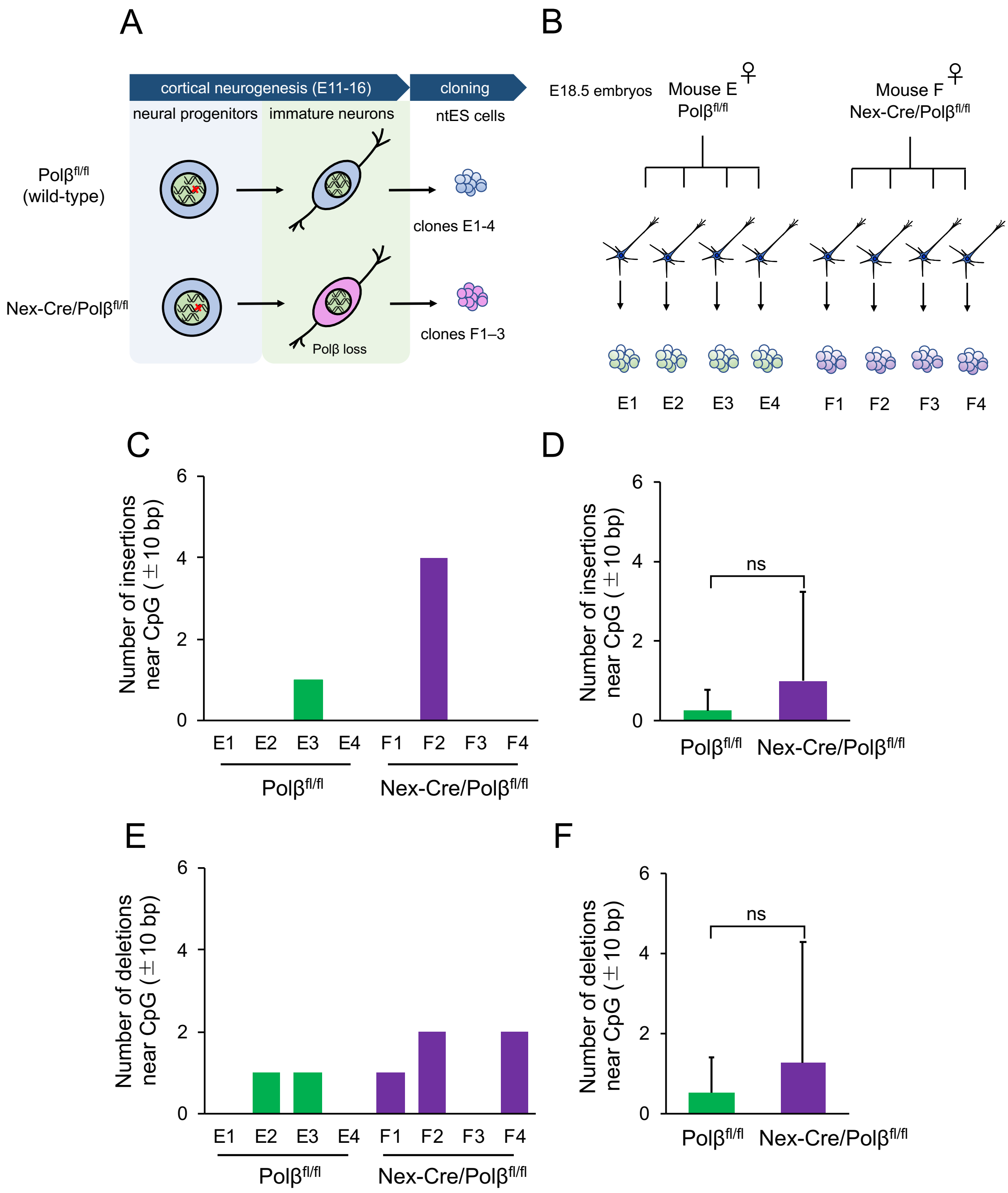

Fig. S4. (**A**) Experimental design for investigating somatic mutation mechanisms during neurogenesis and neuronal differentiation in the cerebral cortex using Nex-Cre/Polβ$^{fl/fl}$ mice. (**B**) Schematic representation of E18.5 embryos and the corresponding ntES cell lines derived from cortical neurons in each mouse. For Nex-Cre/Polβ$^{fl/fl}$ lines, non-littermate Nex-Cre/Polβ$^{fl/fl}$ and Polβ$^{fl/fl}$ embryos were used. (**C**) Number of insertions near CpG sites (within ±10 bp) of the insertion sites in each Nex-Cre/Polβ$^{fl/fl}$ and Polβ$^{fl/fl}$ ntES cell. (**D**) Mean number of insertions near CpG sites (within ±10 bp) in Nex-Cre/Polβ$^{fl/fl}$ and Polβ$^{fl/fl}$ ntES cells. (**E**) Number of deletions near CpG sites (within ±10 bp) of the deletion sites in each Nex-Cre/Polβ$^{fl/fl}$ and Polβ$^{fl/fl}$ ntES cell. (**F**) Mean number of deletions near CpG sites (within ±10 bp) in Nex-Cre/Polβ$^{fl/fl}$ and Polβ$^{fl/fl}$ ntES cells.

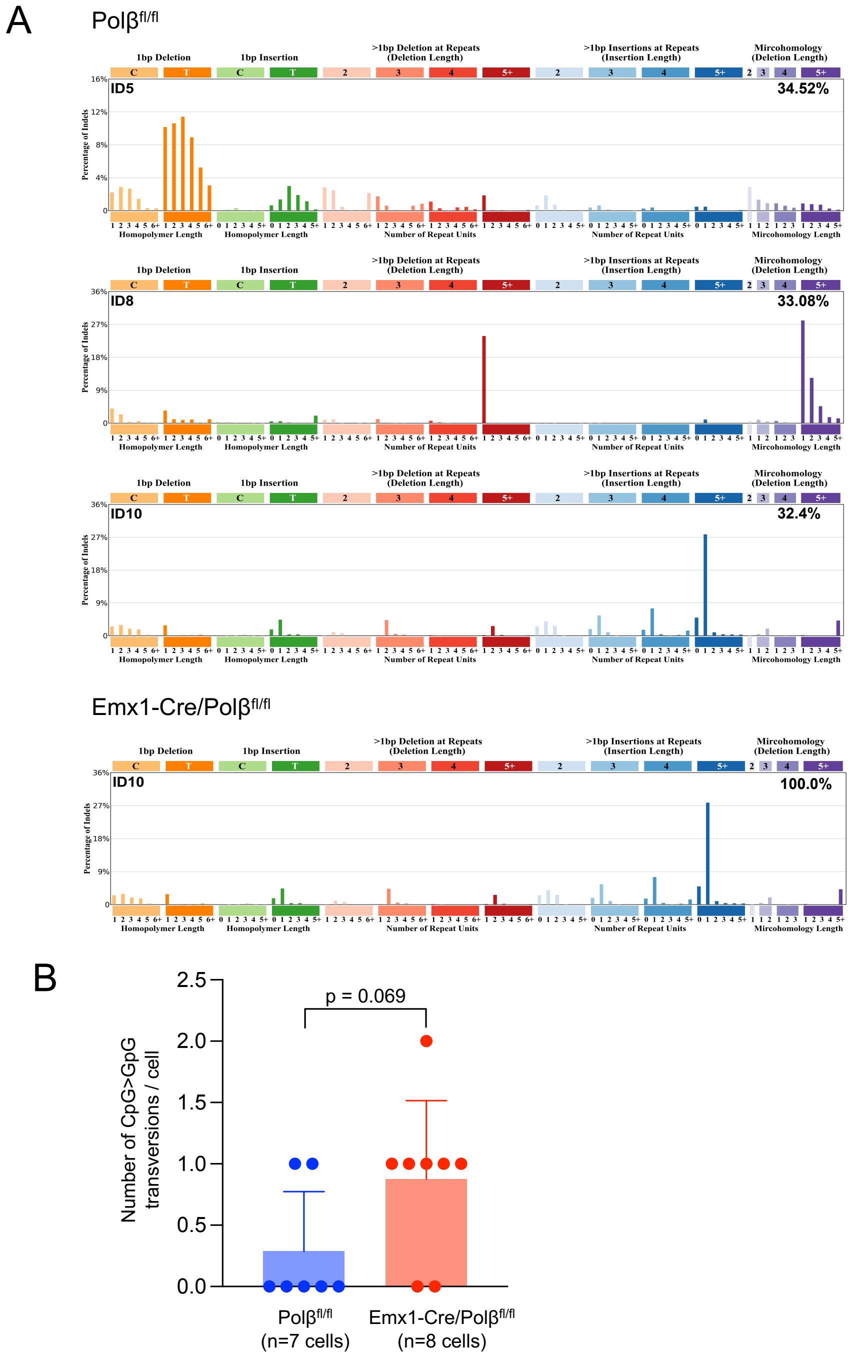

Fig. S5. (**A**) Contribution of COSMIC ID signatures to indels detected in non-repeat sequences of Emx1-Cre/Pol$\beta^{fl/fl}$ and Pol$\beta^{fl/fl}$ ntES cells. (**B**) Mean number of CpG>GpG transversions in Emx1-Cre/Pol$\beta^{fl/fl}$ and Pol$\beta^{fl/fl}$ ntES cells.

clone	chrome	start	end	gene
A2	chr4	7096768	7102035	intergenic (Tox3, Gm33462)
B3	chr7	135933217	135973764	intergenic (Gm45440, Gm36737)
B3	chr2	12092592	12134722	Itga8
C2	chr3	89549539	89551097	Kcnn3
C2	chr3	89551097	89554243	Kcnn3
C2	chr9	35139239	35139307	Dcps
C2	chr19	24139221	24143716	Tjp2
C3	chr2	12664094	12682817	intergenic (Mindy3, Pter)
C4	chr1	118300114	118301780	Tsn
C4	chr8	116378084	116379084	intergenic (1700018P08Rik, Dynlrb2)
C4	chr8	116382013	116387984	intergenic (1700018P08Rik, Dynlrb2)
C4	chr11	69897561	69905058	Neurl4
C4	chr12	8861644	8867022	intergenic (Scd1, 9930038B18Rik)
D1	chr3	130275164	130286753	Col25a1
D1	chr5	64820253	64820303	Klf3
D2	chr3	67668711	67697745	intergenic (Mfsd1, Gm35407)
D2	chr11	100204082	100204613	Krt14
D2	chr11	100205559	100207463	Krt14
D3	chr12	3855403	3877411	Dnmt3a
D3	chr12	68520158	68613615	intergenic (Mdga2, Gm31307)
D4	chr3	78518623	78518882	intergenic (Gm36288, Gm36314)
D4	chr10	118030694	118035603	Gm32663

Fig. S6. Details of SVs detected in Emx1-Cre/Polβ$^{fl/fl}$ and Polβ$^{fl/fl}$ ntES cells.

clone	chrome	start	type	gene
A3	chr3	73905751	LINE	intergenic (Bche, Gm20356)
A3	chr3	149649308	LINE	Gm31121
B2	chr8	67915075	SINE	Psd3
B2	chr13	101247932	LINE	Gm36427
B3	chr2	110828489	LINE	Ano3
B3	chr12	47223693	LINE	intergenic (Stxbp6, Nova1)
B3	chr13	116586662	LINE	intergenic (Itga1, Isl1)
B3	chr16	38522161	SINE	Timmdc1
B3	chr17	40848889	LINE	intergenic (Esp4, Gm6084)
B4	chr1	106904393	LINE	intergenic (Serpinb5, Serpinb12)
C1	chr12	88784873	LINE	Nrxn3
C2	chr1	143592946	LINE	integenic (Gm37796, Cdc73)
C2	chr4	132130578	LINE	Oprd1
C3	chr8	17932519	LINE	intergenic (Csmd1, Gm25665)
C3	chr11	77003910	SINE	Slc6a4
C4	chr2	57088679	LINE	intergenic (Mir195b, Nr4a2)
C4	chr3	157916176	SINE	Cth
C4	chr4	124890906	SINE	Epha10
C4	chr11	84674759	SINE	intergenic (Lhx1, 1700109GRik)
C4	chr13	90305415	LINE	intergenic (Gm37054, Gm47520)
D2	chr16	21547193	LINE	Vps8
D4	chr14	117040543	SINE	Gpc6
D4	chr16	26683448	LINE	Il1rap

Fig. S7. Details of MEIs detected in Emx1-Cre/Polβ$^{fl/fl}$ and Polβ$^{fl/fl}$ ntES cells.

Chromosome	Reference genome size (bp)	Reference genome size except gap regions (bp)	EWC regions (bp)	Proportion of ECW regions (%)
1	195,471,971	191,909,192	161,241,049	84.0%
2	182,113,224	178,326,651	149,987,025	84.1%
3	160,039,680	156,398,855	130,994,998	83.8%
4	156,508,116	152,055,611	123,922,222	81.5%
5	151,834,684	147,919,674	121,555,825	82.2%
6	149,736,546	146,336,543	122,185,471	83.5%
7	145,441,459	141,855,407	110,248,845	77.7%
8	129,401,213	125,611,432	104,784,836	83.4%
9	124,595,110	121,157,018	103,881,801	85.7%
10	130,694,993	127,067,662	107,233,884	84.4%
11	122,082,543	118,745,945	100,770,353	84.9%
12	120,129,022	116,922,420	93,001,400	79.5%
13	120,421,639	117,121,193	96,534,947	82.4%
14	124,902,244	121,442,110	94,330,323	77.7%
15	104,043,685	100,653,315	85,026,207	84.5%
16	98,207,768	95,019,758	80,350,832	84.6%
17	94,987,271	91,707,462	75,849,648	82.7%
18	90,702,639	87,452,634	74,364,209	85.0%
19	61,431,566	58,205,856	49,696,831	85.4%
Total	2,462,745,373	2,395,908,738	1,985,960,706	82.9%

Fig. S8. Effective whole-genome coverage (EWC) regions across each autosome of ntES cells obtained from Emx1-Cre/Polβ$^{fl/fl}$ mouse lines.

Chromosome	Reference genome size (bp)	Reference genome size except gap regions (bp)	EWC regions (bp)	Proportion of EWC regions (%)
1	195,471,971	191,909,192	157,160,112	81.9%
2	182,113,224	178,326,651	146,055,186	81.9%
3	160,039,680	156,398,855	126,836,013	81.1%
4	156,508,116	152,055,611	120,794,458	79.4%
5	151,834,684	147,919,674	118,591,582	80.2%
6	149,736,546	146,336,543	118,302,814	80.8%
7	145,441,459	141,855,407	106,737,673	75.2%
8	129,401,213	125,611,432	102,070,143	81.3%
9	124,595,110	121,157,018	100,736,045	83.1%
10	130,694,993	127,067,662	104,460,986	82.2%
11	122,082,543	118,745,945	99,103,003	83.5%
12	120,129,022	116,922,420	90,435,191	77.3%
13	120,421,639	117,121,193	94,715,288	80.9%
14	124,902,244	121,442,110	92,852,340	76.5%
15	104,043,685	100,653,315	82,964,996	82.4%
16	98,207,768	95,019,758	77,458,310	81.5%
17	94,987,271	91,707,462	73,758,789	80.4%
18	90,702,639	87,452,634	72,643,585	83.1%
19	61,431,566	58,205,856	48,484,690	83.3%
Total	2,462,745,373	2,395,908,738	1,934,161,204	80.7%

Fig. S9. Effective whole-genome coverage (EWC) regions across each autosome of ntES cells obtained from Nex-Cre/Polβ$^{fl/fl}$ mouse lines.

Dataset S1 (separate file). List of somatic SNVs, including their VAFs, in ntES cells (clones A1-3, B1-4, C1-4, and D1-4).

Dataset S2 (separate file). List of somatic indels, including their VAFs, in ntES cells (clones A1-3, B1-4, C1-4, and D1-4).

Dataset S3 (separate file). List of somatic indels at CpG sites, including their VAFs, in ntES cells (clones E1-4 and F1-4).
